# Conservation, Regeneration and Genetic Stability of Regenerants from Alginate-Encapsulated Shoot Explants of *Gardenia jasminoides* Ellis

**DOI:** 10.3390/polym13101666

**Published:** 2021-05-20

**Authors:** Stefanos Hatzilazarou, Stefanos Kostas, Theodora Nendou, Athanasios Economou

**Affiliations:** Department of Horticulture, School of Agriculture, Aristotle University, 54124 Thessaloniki, Greece; skostas@agro.auth.gr (S.K.); dora.nendou@gmail.com (T.N.); aseconom@agro.auth.gr (A.E.)

**Keywords:** artificial seeds, bead hardness, clonal fidelity, gardenia, ISSR, in vitro culture, molecular markers, micropropagation, synseeds

## Abstract

The present study demonstrates the potential of the alginate encapsulation of shoot tips and nodal segments of *Gardenia jasminoides* Ellis, the short-term cold storage of artificial seeds and subsequent successful conversion to desirable, uniform and genetically stable plantlets. Shoot tips and first-node segments below them, derived from shoots of in vitro cultures, responded better than second-to-fourth-node segments on agar-solidified Murashige and Skoog (MS) nutrient medium and thus, they were used as explants for alginate encapsulation. Explant encapsulation in 2.5% sodium alginate in combination with 50 mM of calcium chloride resulted in the production of soft beads, while hardening in 100 mM of calcium chloride formed firm beads of uniform globular shape, suitable for handling. The addition of liquid MS nutrient medium in the sodium alginate solution doubled the subsequent germination response of the beads. The maintenance of alginate beads under light favored their germination response compared to maintenance in darkness. Encapsulated shoot tip explants of gardenia, which were stored at 4 °C for 4, 8 or 12 weeks, showed a gradual decline in their regeneration response (73.3, 68.9, 53.3%, respectively), whereas, non-encapsulated explants (naked), stored under the same time durations of cold conditions, exhibited a sharp decline in regeneration response up to entirely zeroing (48.9, 11.1, 0.0%, respectively). Shoots, derived from 12-week cold-stored encapsulated explants, were easily rooted in solid MS nutrient medium with the addition of 0.5 μM of Indole-3-acetic acid (IAA) and after transplantation of the rooted plantlets individually to pots containing a peat–perlite (3:1, *v*/*v*) substrate, they were successfully acclimatized in the greenhouse under the gradual reduction of 75 or 50% shading with survival rates of 95–100%. The genetic stability of the acclimatized plantlets was assessed and compared with the mother plant using inter simple sequence repeat (ISSR) markers. ISSR analysis confirmed that all regenerated plantlets were genetically identical to the mother plant. This procedure of artificial seed production could be useful for the short-term storage of germplasm and the production of genetically identical and stable plants as an alternative method of micropropagation in *Gardenia jasminoides*.

## 1. Introduction

*Gardenia jasminoides* Ellis, which belongs to the Rubiaceae family, is an evergreen small flowering shrub with dense branches and dark green shiny leaves. The white of the double form flowers, 5–7 cm in diameter, are among the most strongly fragrant of all flowers [1]. Gardenia prefers an acidic soil with a pH of 5.0–6.5 and thrives best in warm temperatures above 5 °C, in humid environments [2]. It is widely used as a garden plant in warm, temperate and subtropical gardens, as well as an ornamental flowering pot plant [3,4]. For the flower industry in Greece, gardenia culture is of great commercial importance with the vast majority of the produced pot plants to be exported to flower markets of the European Union and other countries [4]. The propagation of gardenia can be carried out by seeds, but commercially it is propagated by softwood shoot cuttings in nurseries [1,5,6]. Additionally, various in vitro propagation protocols for *Gardenia jasminoides* have been developed for the production of abundant and uniform plant material for commercial use [7,8,9,10,11,12,13]. However, as far as we know, there are no reports on the artificial seed production of *Gardenia jasminoides*, which could facilitate short- and medium-term cold storage for future propagation.

Artificial or synthetic seeds are defined as explants that are artificially encapsulated in a gel capsule, forming spherical beads [14,15]. The encapsulated explants (shoot tips, nodal segments, axillary buds, etc.) upon germination behave like seeds and possess the ability to convert into plantlets under in vitro or in vivo conditions [16,17]. The encapsulation technique mimics that of the natural seed technology and combines the advantages of seed propagation with those of clonal propagation [18,19,20]. Thus, this method is an important application of in vitro culture and also plays a significant role for short- and medium-term germplasm storage contributing to large scale propagation in the nurseries [21,22]. The genetic uniformity, in most cases, is maintained between regenerated plantlets and mother plant donors of the encapsulated explants [23]. Elite genotypes, in the form of artificial seeds, are easily preserved under cold storage conditions and can be regenerated later upon demand for planting material [24,25]. Furthermore, the method offers the easy handling of artificial seeds during storage and is unhindered from custom barriers distribution and exchange from one country to another, while it is cost efficient compared to the traditional in vitro technique, avoiding frequent subcultures [14,26,27,28,29]. One of the most important factors for the successful use of the encapsulation technique is the proper gel material. Many different agents for artificial seed production were used in the past of various effectiveness [30,31]; however, the most common in use so far with optimal results on explant encapsulation is the alginate matrix, due mainly to fast gelation, weak spinability of the solution, sensible thickness and low cost [32,33,34]. Alginate is a natural biopolymer derived from brown seaweeds. It exists in the market as a sodium salt, sodium alginate, with the ability to form gels in the presence of calcium ions which are able to encapsulate small plant tissues and organs and protect them from mechanical damage and desiccation, allowing germination and conversion to occur [30,31,33]. The successful application of the encapsulation method for artificial seed production has been reported for many plant species, and most recently for the woody plants of *Rosa hybrida* [35], *Viburnum dentatum* [29], *Albizia lebbeck* [36] and *Citrus jambhiri* [37].

In some cases, the plantlets derived from any kind of in vitro cultures may exhibit genetic variation due to many reasons, such as the type of explant source and genotype, repeated subcultures, cold storage or slow growth practices and other physical and chemical factors [24,38,39]. Therefore, the genetic uniformity of the regenerants from artificial seeds should be examined prior to any commercial exploitation. To evaluate the genetic stability of micropropagated plantlets, various DNA techniques have been employed, including amplified fragment length polymorphism (AFLP), random amplified polymorphic DNA (RAPD), simple sequence repeat (SSR) and ISSR [40,41]. Among them, the most frequently used are RAPD and ISSR since they are simple, fast, highly discriminative, reliable, widely available, offer easily handling and are cost-efficient [23,42,43,44]. ISSR markers have been successfully applied to detect genetic variation between regenerants from cold-stored artificial seeds and mother plants from which the explants were taken in *Rauvolfia tetraphylla* [23], *Rauvolfia serpentina* [43], *Sphagneticola calendulacea* [24], *Albizia lebbeck* [36], *Viburnum dentatum* [29], *Hedychium coronarium* [45] and other plant species.

The objective of the current work was to develop an efficient procedure for artificial seed formation, by using shoot explants encapsulated in alginate matrix, aiming at establishing an alternative micropropagation method for production of commercial planting material for gardenia pot plant cultivation. Thus, preparation of artificial seeds through alginate encapsulation, their short-term cold storage and subsequent germination followed by an assessment of genetic uniformity of regenerants using ISSR markers were investigated.

## 2. Materials and Methods

### 2.1. Plant Material and In Vitro Establishment of Explants

Apical shoots were collected from a single gardenia (*Gardenia jasminoides* Ellis cv. Pelion) plant, grown in a 10 L container as the mother plant for cuttings in the greenhouse plant collection of the Experimental Farm of the Aristotle University, Thessaloniki, Greece. From these shoots, shoot tip explants (1.0–1.5 cm long) were excised and immersed in a solution of 0.1% (*w*/*v*) mercuric chloride (HgCl_2_) for 5 min and subsequently surface-disinfested in 2.0% (*v*/*v*) sodium hypochlorite (NaOCl) containing 0.05% (*v*/*v*) Tween-20 for 5 min with continuous shaking in a magnetic stirrer. The explants were then rinsed three times under aseptic conditions in sterile distilled water to remove any trace of sterilants. Afterwards, each explant was inoculated in a single glass tube (100 × 25 mm) containing 10 mL of MS nutrient medium [46], supplemented with 5 μM of 6-Benzylaminopurine (BAP) (Sigma-Aldrich, St. Louis, MI, USA), 2% (*w*/*v*) sucrose and 0.7% (*w*/*v*) agar (Technobiochem, Athens, Greece) as a gelling agent. The nutrient medium pH was adjusted to 5.8 before agar addition and the glass tubes sealed with aluminum foil were autoclaved at 121 °C and 122 kPa for 20 min. After six weeks of culture, multiple shoots were formed, which were used for the encapsulation experiments.

### 2.2. Explant Determination for Encapsulation

For the selection of the most suitable type of explant for encapsulation, shoot tips (3–4 mm long) and equal size nodal segments with axillary buds, from the 1st, 2nd, 3rd or 4th node below the shoot tip, were dissected from the previously in vitro established gardenia cultures and tested for their capacity on shoot regeneration. The explants were individually transferred to glass tubes containing 10 mL of solid MS nutrient medium supplemented with 5 μM BAP. The frequency (%) of explants producing shoots, number of shoots and shoot length per explant were recorded after four weeks of culture. Based on these measurements, shoot tips and 1st-node segments were chosen and used, in equal numbers, as the most responding to shoot induction explants for the production of artificial seeds, unless otherwise specified.

### 2.3. Encapsulation and Artificial Seed Germination

To evaluate the inclusion of MS nutrient medium in the encapsulation matrix on subsequent shoot regeneration, shoot tip and 1st-node segment explants (in equal number) were immersed in a sterile solution of 2.5% sodium alginate (control) (molecular weight 195.16 (theoretical) or 219 (actual average); AppliChem, Darmstadt, Germany)) and also in autoclaved MS liquid nutrient medium (without Ca) supplemented with 2.5% sodium alginate as the gelling agent. Then, explants from the two treatments were taken by forceps and dropped individually to two different beakers containing a sterile solution of 50 mM calcium chloride (CaCl_2_^.^2H_2_O) under gentle stirring for 30 min, in order to complete the ion-exchange (Na^+^/Ca^2+^) reaction. All operations were conducted in the sterile conditions of the laminar-flow hood, at a room temperature of 23 °C. After hardening, the produced beads were retrieved and rinsed twice with sterile distilled water to remove traces of calcium chloride. In order to test the recovery of shoots under in vitro conditions, the beads were transferred to glass tubes (100 × 25 mm) containing 10 mL of solid MS nutrient medium supplemented with 1 μM of IAA (Sigma-Aldrich, St. Louis, MI, USA) and 5 μM of BAP and incubated in a growth chamber under light or in darkness provided by covering the cultures with a black fabric sheet. The evaluation of the regeneration response was made after four weeks of culture by estimating the shoot formation frequency (%) and measuring the number and length of produced shoots.

The calcium chloride concentration as complexing agent for optimal bead formation was also evaluated by dipping separately, with forceps, the gardenia explants (shoot tips and 1st-node segments in equal number) coated in a 2.5% sodium alginate into 25, 50, 100 or 200 mM calcium chloride solution where they remained for 30 min with slow agitation to accomplish bead formation. Afterwards, the beads were rinsed with sterile distilled water and transferred individually to glass tubes containing solid MS nutrient medium and placed in the growth chamber under light, as described previously. The evaluation of the regeneration response of the encapsulated explants took place after four weeks of culture by taking the same measurements as mentioned in the previous experiment. For the instrumental analysis of the hardness of the beads, which were produced under the four different concentrations of calcium chloride, a Texture Analyzer TA XT2i (Stable Microsystems, Godalming, Surrey, UK) was used. Thus, each bead was placed on the ‘crisp fracture support ring’; a flat aluminum plate (75 mm diameter) connected to the analyzer was used. The force required for a 0.2 mm deformation of the bead capsule was recorded. The speed of the compression plunger was 1 mm s^−1^. For each calcium chloride concentration, 10 beads were used.

### 2.4. Cold Storage of Artificial Seeds

For cold conservation, encapsulated (beads) and non-encapsulated gardenia shoot tip explants were maintained in a cold room at 4 °C for 4, 8 or 12 weeks. The beads were produced in 2.5% sodium alginate prepared in liquid MS nutrient medium and thereafter hardened in 100 mM calcium chloride for 30 min, at room temperature of 23 °C, as previously described. They were placed then on filter paper moistened with sterile distilled water in sealed Petri dishes and stored at 4 °C in darkness. The non-encapsulated explants (naked) were cultured on growth regulator-free solid MS nutrient medium in glass tubes maintained at 4 °C under light. After each cold storage period, both beads and non-encapsulated explants were transferred onto fresh solid MS nutrient medium in glass tubes with the addition of 1 μM IAA and 5 μM BAP plus 2% sucrose and maintained in the growth chamber under standard temperature and light conditions as previously described for in vitro cultures. Their regrowth response was recorded after four weeks of culture.

### 2.5. Rooting of Shoots and Acclimatization of Plantlets

The evaluation of shoot rooting and plantlet acclimatization ability was conducted by using shoots regenerated from encapsulated shoot tips of gardenia after their cold storage (4 °C) for 12 weeks. Regenerated individual shoots were transferred aseptically for root induction into glass tubes (100 × 25 mm) containing 10 mL of agar-solidified (7%, *w*/*v*) MS nutrient medium supplemented with 0.5 μM IAA and 2% (*w*/*v*) sucrose. After four weeks, the rooted shoots (plantlets) were washed gently under running tap water for the removal of any medium traces and then moved to the greenhouse and transplanted to 1 L plastic pots containing a mixture of 1:1 (*v*/*v*) peat TS2 Klasmann (Klasmann-Deilmann, Geeste, Germany) and perlite (Isocon, Athens, Greece). Afterwards, the pots were placed in groups of 25 plantlets under polyethylene net covers of different degrees of shading (25%, 50% or 75%) on a bench, equipped with an electronically controlled water-fogging system, for acclimatization. Under 25% shading, the plantlets remained for one or two weeks and then transferred to full-light irradiance (approx. 330 μmol m^−2^ s^−1^) for three or two weeks, respectively. In the treatment of 50% shading, the plantlets were kept in these conditions during the 1st week, then moved under 25% shading for one more week and finally transferred to full-light irradiance. The third treatment was plantlet placement for one week under 75% shading followed by one week under 50% shading and another week under 25% shading before transferring under full-light irradiance. The survival rate (%) of gardenia plantlets for all treatments was recorded at the end of the 4-week acclimatization. The relative humidity (RH) under the polyethylene net covers decreased gradually from approximately 95% (when 75% shading was applied) to 85% (under 50% shading) down to 75%, under 25% shading. Additionally, the light irradiance (photosynthetic photon flux density (PPFD)) measured at plantlet level was 135 μmol m^−2^ s^−1^ under 75% shading, 170 μmol m^−2^ s^−1^ under 50% shading, 205 μmol m^−2^ s^−1^ under 25% shading and 330 μmol m^−2^ s^−1^ under full-light irradiance. During acclimatization, the natural photoperiod in the greenhouse was 12–13 h and the ambient temperature 24 ± 2 °C. After acclimatization, successfully established plantlets of gardenia were kept in the greenhouse for normal growth and development.

### 2.6. Environmental Conditions of In Vitro Cultures

The in vitro cultures of all experiments were maintained in a CRW-500SD growth chamber (Chrisagis, Athens, Greece) with the temperature set at 23 ± 0.1 °C and the relative humidity (RH) at 65 ± 1%. The photoperiod was 16/8 h (light/dark) at a PPFD of 52 μmo m^−2^ s^−1^ at culture level from cool-white fluorescent tubes.

### 2.7. Genetic Stability Assessment Using ISSR Markers

Young leaves were collected from eight randomly selected and acclimatized plantlets, which were produced from the regeneration of encapsulated gardenia shoot tips after a 12-week cold storage, and the mother control plant from which the explants were taken and used for genetic analysis. They were lyophilized and used for DNA extractions with NucleoSpin Plant ΙΙ kit (Macherey Nagel, Düren, Germany) according to the instructions of the package. The quality and quantity of genomic DNA (gDNA) was assessed by electrophoresis of gDNA by using a NanoDrop™ 2000 Spectrophotometer (Thermo Electron Corporation, Waltham, MA, USA). PCR amplifications were performed in a volume of 20 μL using 40 ng total DNA, Horse-Power™ Taq DNA Polymerase MasterMix (Canvax Biotech, Cordoba, Spain), which contained all PCR reaction components (Taq Polymerase, PCR buffer, dNTPs and Mg^2+^) and 1 μM from each primer. Twenty ISSR primers were initially tested, and based on preliminary work, ten of them (UBC 808, 809, 810, 811, 815, 816, 818, 821, 834 and 841) (University of British Columbia, Vancouver, BC, Canada), which gave reproducible results, were selected for further use. Amplifications were carried out by a chain of processes: an initial denaturation step at 94 °C for 4 min, 35 cycles comprising a denaturation cycle at 94 °C for 1 min, 1 min annealing at specific temperature for each primer, annealing at 72 °C for 2 min and a final extension step at 72 °C for 10 min. PCR amplifications were performed on SimpliAmp^TM^ Thermal Cycler (Life Technologies, Thermo Fischer Scientific, Waltham, MA, USA). Amplification products were separated on 1.4% agarose gel using 1 × TAE buffer (Tris, acetic acid and EDTA) and stained with ethidium bromide. The gels were photographed under UV using a gel documentation system Transilluminator UV light (Biostep, Burkhardtsdorf, Germany). Two independent PCR amplification reactions were performed for each sample. All the ISSR bands were compared with each other for all the DNA samples to ascertain the genetic similarity of the plant material.

### 2.8. Statistical Analysis

The experiments were conducted using completely randomized designs. For the plantlet acclimatization, 20 replicates per treatment were used, while in all other experiments, 45 replicates per treatment were employed. The statistical analysis of the data was based on analysis of variance (ANOVA). Data in percentages were subjected to arcsin transformation for proportions prior to statistical analysis. Duncan’s multiple range test was applied to distinguish statistically significant differences among means at *p* ≤ 0.05. The statistical analysis was performed using the SPSS 23 software (SPSS Inc., Statistical Package for the Social Sciences, Chicago, IL, USA).

## 3. Results and Discussion

### 3.1. Explant Determination for Encapsulation

**Figure 1:**
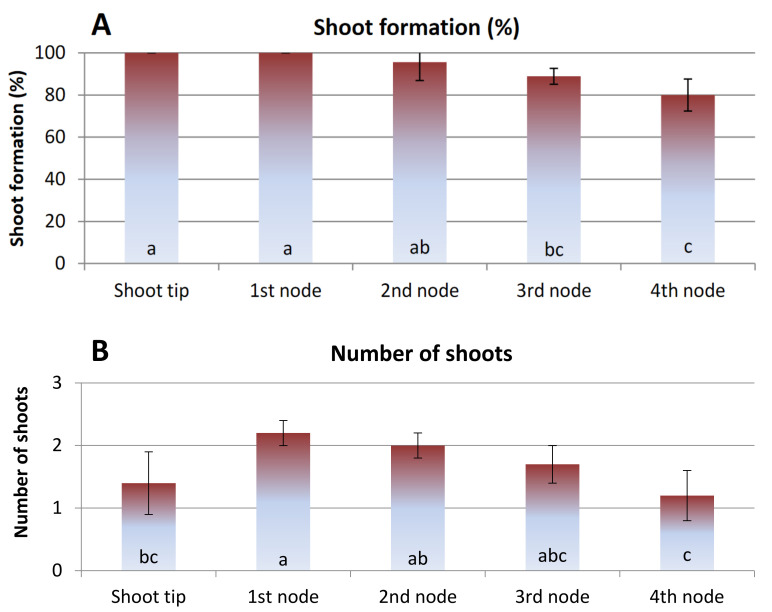

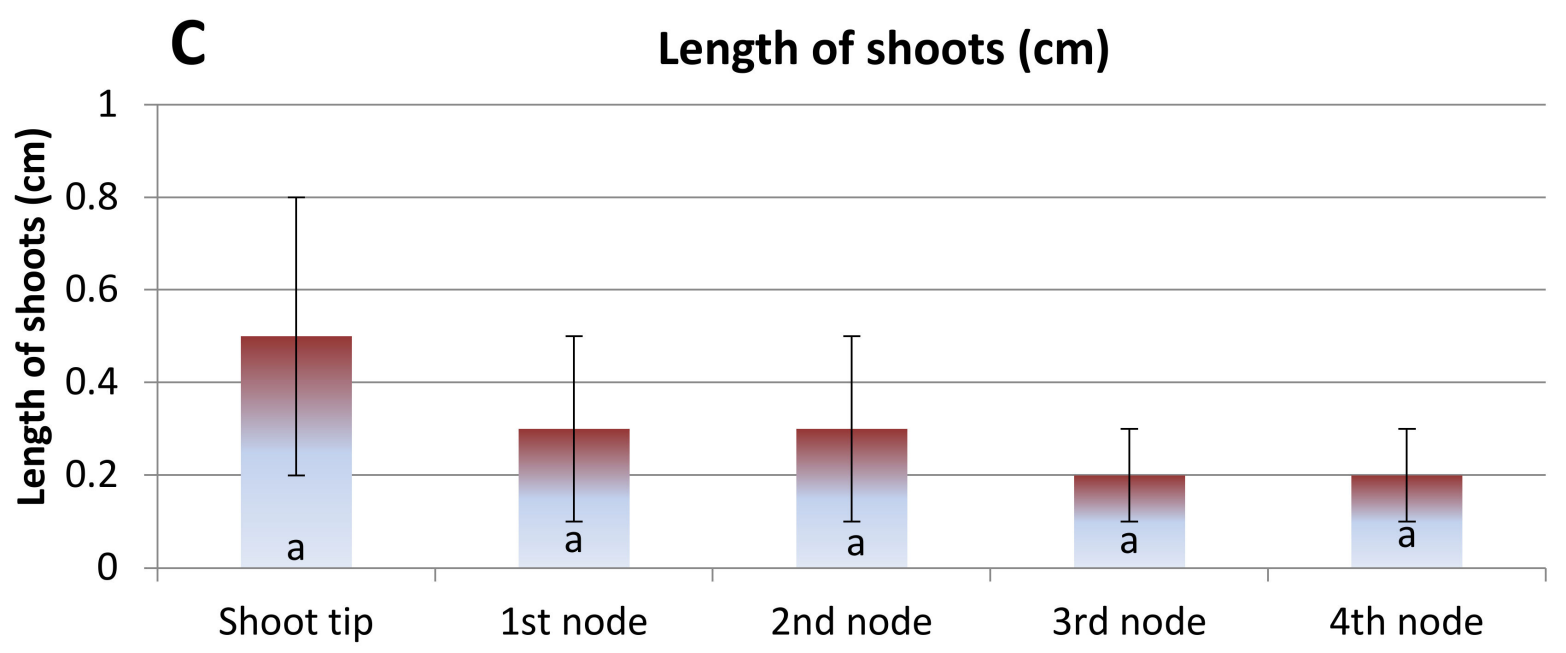
Effect of explant type on (**A**) shoot formation frequency (%), (**B**) number and (**C**) length of shoots of *G. jasminoides* cultured on agar-solidified MS nutrient medium supplemented with 5 μM of BAP, after 4 weeks of culture. Bars represent the means ± SD (*n* = 45). Bars with different letters are significantly different according to Duncan’s multiple range test at *p* ≤ 0.05.

The type of explant significantly influenced shoot formation frequency (%) and the number of shoots produced, while no statistical differences were noticed for the length of shoots. Thus, shoot tip and first-node explants, excised from in vitro cultures, sprouted at frequencies of 100% followed by second-node explants at a frequency of 96% (Figure 1A). Significantly lower shoot formation frequencies were found in the explants of the third- and fourth-node segments (89% and 80%, respectively) than in shoot tip and first-node explants. The highest number of shoots was counted in the first-node explants, while the longest shoots in the shoot tip explants did not have significant differences compared to the other types of explants (Figure 1B,C). These results justify the choice of shoot tip and first-node explants, excised from shoots produced in vitro, as the most suitable explant source for encapsulation.

The type of explant used for encapsulation is crucial for short- to medium-term storage of artificial seeds and successful regrowth later during germination. For most woody plant species, shoot tips, nodal segments and individual axillary buds have been employed for encapsulation with different degree of success [17,18,22,47,48]. In the present study, shoot tip and first-node gardenia explants exhibited higher response in vitro than the other nodal segments and thus, they were chosen as potential explants for alginate encapsulation. This is probably due to the higher juvenility of their tissues and consequently they have greater proliferation ability during artificial seed germination. The suitability of shoot tips and/or nodal segments as the explant source for encapsulation leading to artificial seed production has also been reported for several woody plant species; some of them are: *Punica granatum* [49], *Nerium oleander* [47,50], *Simmondsia chinensis* [51], *Cassia angustifolia* [27], *Citrus sinensis* [52], grapevine rootstock ‘Kober 5BB’ [19], *Olea europaea* [53], *Viburnum dentatum* [29], etc.

### 3.2. Encapsulation and Artificial Seed Germination Assessment

Shoot tip and first-node gardenia explants were adequately encapsulated, forming beads by dipping them into 2.5% sodium alginate solution for coating and then transferring them into 50 mM of calcium chloride for hardening for 30 min. The shoot regeneration response of the beads, irrespective of encapsulation matrix composition, was significantly higher under light than in darkness (Table 1). The inclusion of liquid MS nutrient medium in the alginate solution doubled the shoot regeneration response of the encapsulated explants compared with the MS-free alginate solution both under light germination of beads (88.9% vs. 46.7%) and in darkness (28.9% vs. 13.3%) (Table 1). The same trend was also noticed concerning the number of shoots produced per bead. The number of shoots per germinated bead was higher under light than in darkness. In addition, in both light regimes, the presence of MS nutrient medium in the alginate matrix enhanced shoot proliferation of encapsulated explants (Table 1). Furthermore, the proliferated shoots were longer when bead germination took place under light than in darkness, with the longest shoots being produced in the alginate matrix containing MS nutrient medium (Table 1).

The inclusion of MS nutrient medium in the alginate matrix promoted the shoot regeneration response in gardenia beads and improved shoot formation. It seems that such an alginate matrix enriched with MS nutrient medium, serving as an artificial endosperm, and provided the nutrients that were essential for the growth of the encapsulated explants during the process of their germination. The beneficial effect of MS nutrient medium addition to the alginate matrix on the germinability of the artificial seeds has also been reported for several woody plant species, among them being *Photinia fraseri* [47], *Rauvolfia tetraphylla* [23], *Cassia angustifolia* [27], *Rosa damascena trigintipetala* [54], *Nerium oleander* [50] and *Viburnum dentatum* [29]. The presence of nutrients in the alginate capsule may increase, in addition to germination competence, the viability of the artificial seeds [34] and also their storage capacity [55]. Germination response and shoot proliferation of encapsulated shoot tip and first-node explants of gardenia, after four weeks of culture, were promoted under light conditions (16 h per day) compared to culture in darkness. This is in agreement with an earlier report for *Viburnum dentatum* [50].

**Figure 2:**
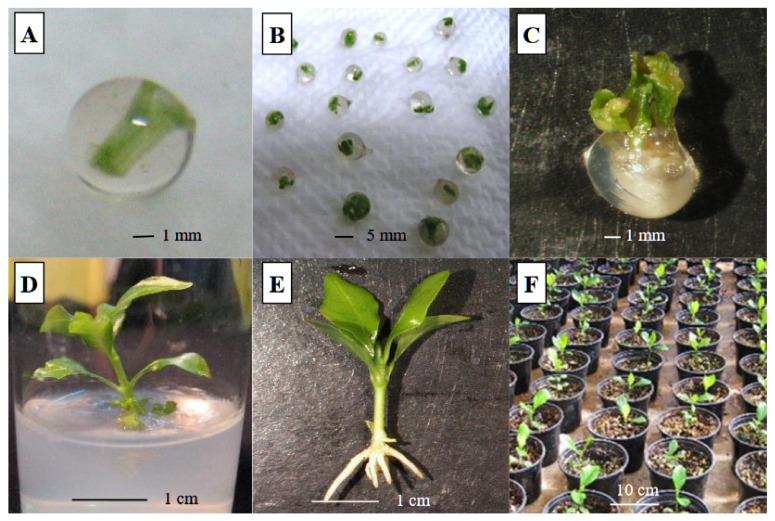
Artificial seed production of *G. jasminoides*, germination after cold storage and conversion into plantlets: (**A**) encapsulated nodal segment in 2.5% sodium alginate followed by hardening in 100 mM calcium chloride for 30 min. (**B**) Artificial seeds during storage at 4 °C. (**C**) Germination of cold-stored (for 12 weeks at 4 °C) artificial seed (encapsulated shoot tip), after 10 days on MS nutrient medium. (**D**) Shoot, derived from germinated artificial seed of cold storage, 10 days after placing for rooting in agar-solidified MS nutrient medium with 0.5 μM IAA. (**E**) Rooted shoot after removal from the agar-solidified MS nutrient medium and before transplanting to peat–perlite substrate. (**F**) Gardenia plantlets established in the greenhouse, after being acclimatized under 75% shading of weekly reduction, 2 weeks from the end of the acclimatization period.

The concentration of calcium chloride, as hardening agent, had a decisive effect on the texture and hardness of beads and the subsequent regrowth of gardenia explants (Table 2; Table 3). Thus, a high concentration of calcium chloride (200 mM) produced very hard (0.37 N), uniform and globular-shaped beads, whereas a low concentration (25 mM) produced very delicate and fragile (0.14 N) beads of indefinite shape. Using 50 mM of calcium chloride, the produced beads were solid and of uniform shape but soft (0.20 N). The best texture of beads was obtained in the treatment of 100 mM calcium chloride, in combination with 2.5% sodium alginate, for 30 min. The produced beads were uniform, firm (0.27 N) of globular shape and suitable for handling (Table 2, Figure 2A). The break of the capsule wall and the emergence of the explants were noticed seven days after placement in vitro for the germination of beads that were formed with the use of 25, 50 or 100 mM calcium chloride, and after ten days for those formed with 200 mM calcium chloride (data not shown). The highest shoot regeneration response was measured on beads hardened with 100 mM calcium chloride (51.5%) and the lowest with 20 mM, while intermediate regeneration responses were found with 50 and 200 mM (40.0% and 42.2%, respectively) (Table 3). Beads formed with 100 mM calcium chloride were more prolific than those of the other concentrations tested, producing 2.7 shoots per explant. The same trend was also noticed concerning the length of shoots, which were bigger than that of the other concentrations except for the 50 mM of calcium chloride (Table 3). The results indicated that the concentration of calcium chloride affected the physical characteristics of the beads, with the concentration of 100 mM proving to be the optimal complexing agent, for satisfactory bead formation and the subsequent germination response of artificial seeds of *G. jasminoides*.

It is known from the literature that the rigidity and permeability of the bead capsule, as well as the bead shape, are greatly influenced by the concentrations of sodium alginate and calcium chloride together with the time of complexation facilitating the completion of ion exchange between Na^+^ and Ca^2+^ [19,26,49,51,56,57,58,59]. The capsule should be firm enough to allow for easy bead handling without breakage, but weak enough to allow for the explant to emerge from the capsule upon germination [56]. This balance between hardness and weakness can be achieved, in most of the cases, by the use of sodium alginate in combination with calcium chloride in different time exposures and concentrations between them [19,33]. Thus, beads can be produced with different shape, texture and transparency according to required needs [28,39]. Attia et al. [54] reported that increasing the sodium alginate concentration from 3 to 5% (*w*/*v*) in combination with 75 or 100 mM calcium chloride delayed the germination of the artificial seeds for one week. In addition, high concentrations of sodium alginate (4 or 5%) or calcium chloride (200 mM), within an ion exchange with a duration of 30 min, resulted in the formation of hard-texture beads and delayed their germination [23]. On the other hand, lower concentrations of sodium alginate (1 or 2%) or calcium chloride (25 or 50 mM) produced weak structures with no define shape which disintegrated while being handled [45,60]. In general, it is concluded that 2–3% sodium alginate gel exposed for 30 min to 100 mM calcium chloride is an optimal combination for acceptable bead production and subsequent satisfactory germination response [29,30,61]. In the present study, the combination of 2.5% sodium alginate with 100 mM of calcium chloride for a 30 min complexation time resulted in the formation of high quality beads, easy to handle (hardness 0.27 ± 0.02 N), followed by an unimpeded germination response. The use for complexation 100 mM calcium chloride for 30 min has been reported for many woody plant species such as *Photinia fraseri* and *Syringa vulgaris* [47], *Simmondsia chinensis* [51], *Rauvolfia tetraphylla* [23], *Acacia* hybrids [31], *Nerium oleander* [47,50], *Terminalia arjuna* [62], grapevine rootstock ‘5BB’ [19], *Celastrus paniculatus* [60], *Viburnum dentatum* [29], and several others.

### 3.3. Post-Cold-Storage Regrowth of Artificial Seeds

**Figure 3:**
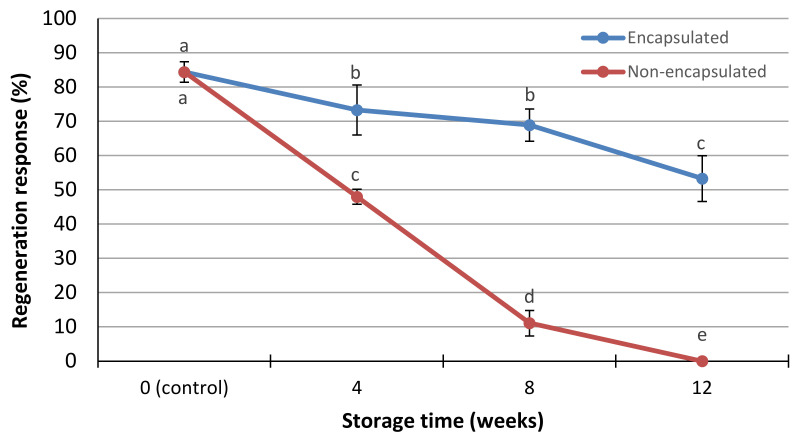
Effect of storage at 4 °C for different time periods (weeks) on subsequent regeneration responses (%) of encapsulated and non-encapsulated shoot tip explants of *G. jasminoides*, on MS nutrient medium with 1 μM IAA and 5 μM BAP, after 4 weeks of culture. Means ± SD (*n* = 45) followed by different letters are significantly different according to Duncan’s multiple range test at *p* ≤ 0.05.

The cold storage of encapsulated (Figure 2B) and non-encapsulated (naked) gardenia shoot tip explants at 4 °C for different time periods significantly influenced their subsequent regeneration response, as recorded four weeks after transferring to fresh MS nutrient medium under the same culture (light and temperature) conditions. When cold storage was not applied (control cultures), the regeneration response was 84.4% for both encapsulated and non-encapsulated explants (Figure 3). However, as the cold storage duration (at 4 °C) increased, the regeneration response decreased gradually. Encapsulated explants kept at 4 °C for 4, 8 or 12 weeks exhibited a gradual but mild decline in their regeneration response, starting from 73.3% when stored for 4 weeks, to 68.9% for 8 weeks and ending to 53.3% after 12 weeks of cold storage (Figure 3). On the contrary, the non-encapsulated (naked) explants showed a drastic decline in their regeneration response, which was 48.0, 11.1 and 0% after 4, 8 and 12 weeks of cold storage, respectively (Figure 3). Furthermore, the encapsulated explants, irrespective of the cold storage duration, produced significantly more shoots after germination than non-encapsulated explants exposed to the same cold storage (2.3 and 1.2 shoots per explant, respectively) (Table 4). Although cold storage caused a marked reduction on shoot length of non-encapsulated explants after their culture under standard conditions, this shoot length, however, was not significantly different from the length of shoots produced from encapsulated explants (Table 4).

It is obvious, from this study, that encapsulated shoot tips of gardenia exhibited higher adaptability at 4 °C storage than non-encapsulated shoot tips. After 12 weeks of storage, the regeneration response (Figure 2C) was 53.3% of encapsulated shoot tips, whereas non-encapsulated shoot tips did not sprout (Figure 3) indicating the complete loss of their viability. The viability and regeneration potential of the encapsulated explants, in respect to non-encapsulated ones, after a short-term cold storage, could be attributed to the gel coating which acts protectively and nutritionally towards the encapsulated explants like an artificial endosperm [19,27,29,63,64,65]. The protective and nutritional role of the alginate matrix, contributing to satisfactory survival and regrowth after cold storage, has been referred to in a number of previous reports in the literature [19,27,29,47,50,52]. During storage at 4 °C of the encapsulated gardenia shoot tip explants, a gradual reduction in the regeneration response was observed of their subsequent cultures, under standard conditions, from 73.3% after 4 weeks storage to 53.3% after 12 weeks of cold storage. This gradual decline may have derived from the obstructed respiration of encapsulated explants, probably due to the protective coating of the alginate matrix or from partial desiccation of explants during cold storage, causing a loss of moisture [19,43,50,66,67,68,69]. Regeneration decline has also been observed after a short-term cold storage of artificial seeds in numerous woody plant species, among them in *Rauvolfia tetraphylla* [70], *Decalepis hamiltonii* [71], *Cassia angustifolia* [27], *Nerium oleander* [47,50], *Citrus sinensis* [52], grapevine rootstock ‘Kober 5BB’ [19], *Rosa damascena trigintipetala* [54], *Celastrus paniculatus* [60] and *Viburnum dentatum* [29].

### 3.4. Rooting of Shoots and Acclimatization of Plantlets

Gardenia shoots developed from encapsulated shoot tip explants, after a 12-week cold storage, were induced to root in vitro (Figure 2D). With the addition of 0.5 μM of IAA to the solid MS nutrient medium, gardenia shoots formed roots in a 4-week rooting period at very high percentages (data not shown), which is in agreement with a previous work of Hatzilazarou et al. [11] referring to gardenia microshoot rooting up to 100% in vitro. These findings showed that the rooting ability of gardenia shoots, which were generated from encapsulated explants after being stored at 4 °C for 12 weeks, was not affected by the cold storage, achieving high rooting percentages. Similar observations on the rooting of shoots derived from regrowing artificial seeds, after cold storage, were reported for *Nerium oleander* [50] and *Viburnum dentatum* [29].

**Figure 4:**
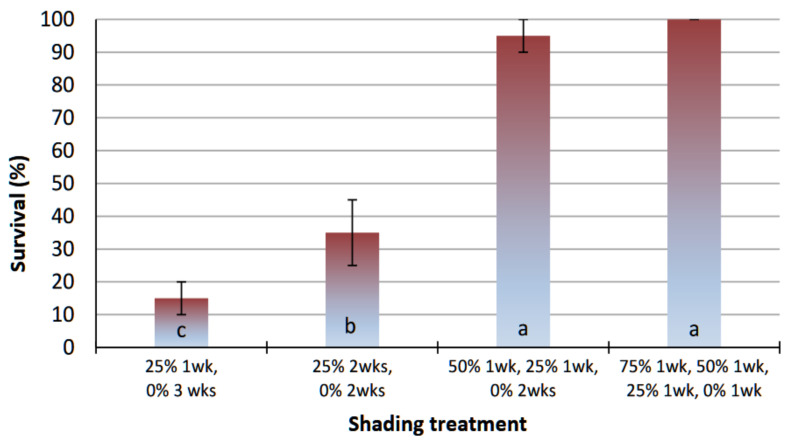
Effect of different shading treatments during acclimatization of in vitro-produced plantlets of *G. jasminoides,* derived from regenerant shoots of encapsulated shoot tip explants which had stored at 4 °C for 12 weeks, on their survival rate. Bars represent the means ± SD (*n* = 45). Bars with different letters are significantly different according to Duncan’s multiple range test at *p* ≤ 0.05.

Rooted shoots (plantlets), after thorough removal from the agar medium avoiding root destruction (Figure 2E), were transplanted to plastic pots filled with a 1:1 (*v*/*v*) peat–perlite mixture and transferred to a greenhouse bench, under different shading conditions, for acclimatization. It was found that the time of shading and degree of shading applied over the young gardenia plantlets markedly affected their acclimatization and subsequent growth. Young plantlets placed after transplanting for one week under 75% shading and then transferred weekly to 50% and 25% shading and finally to direct sun light for an extra week scored a survival rate of 100% (Figure 4). Lower survival rate (95%) was achieved when plantlets were kept under 50% shading in the first week followed by 25% shading in the second week and then moved to direct sun light for two additional weeks. However, when plantlets remained for one or two weeks under 25% shading and were moved to full sun light for the remaining three or two weeks, their survival rate dropped to 15% and 35%, respectively (Figure 4).

Acclimatization is a time-consuming and labor-intensive process contributing to a major portion of the production cost in micropropagation [72]. Thus, the acclimatization stage is maybe the most crucial for the successful micropropagation of plant species [73,74]. This is due to the fact that there are enormous differences between in vitro conditions and greenhouse conditions. It is known that in in vitro conditions, the propagules are cultured in test tubes or vessels under aseptic conditions, low light irradiance and high relative humidity [75]. Besides, the culture medium contains nutrients and sugar, and thus the propagules are usually grown heterotrophically since any roots formed are not always functional due to absence of root hairs [74,76]. The results of this study showed that the adaptation of young plantlets to the new environment should be carried out gradually and carefully. It could be said that the environmental conditions under high shading during acclimatization were more or less similar to those of the in vitro environment. Thus, the young plantlets at first were kept under low light irradiance and high relative humidity and then gradually the light irradiance increased, and the relative humidity decreased, resulting in a successful acclimatization process and further satisfactory establishment and survival in the greenhouse environment (Figure 2F). The gardenia plantlets that survived after acclimatization did not have any visible variation in respect to morphology and/or growth characteristics. The successful implementation of acclimatization to plantlets originated from germinated artificial seeds have also been reported for the woody plant species of *Punica granatum* [49], *Simmondsia chinensis* [51], *Rauvolfia tetraphylla* [23], *Rauvolfia vomitoria* [77], *Nerium oleander* [50], *Citrus jambhiri* [37], *Albizia lebbeck* [36] and *Viburnum dentatum* [29].

### 3.5. Genetic Stability Assessment Using ISSR Markers

**Figure 5:**
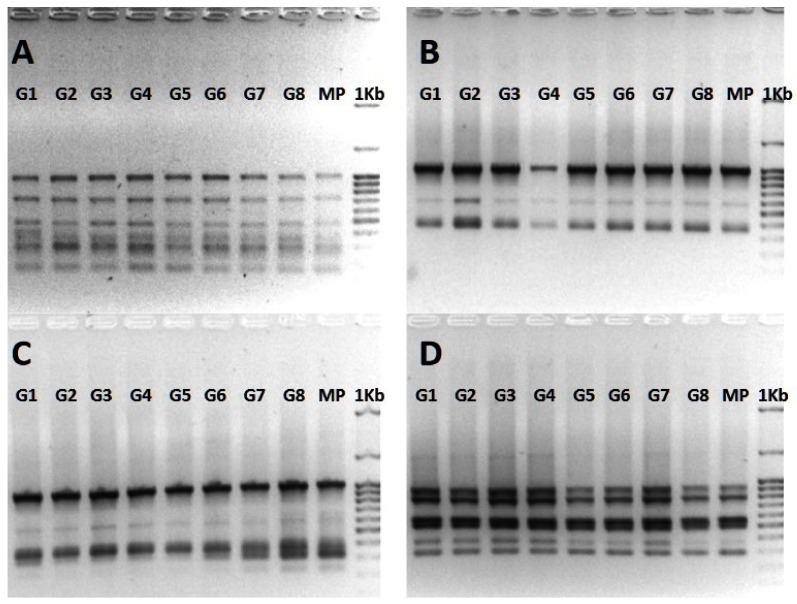
ISSR amplification profiles of eight plantlets (G1–G8), derived from cold-stored germinated artificial seeds, and mother plant (MP) of *G. jasminoides* obtained with primers UBC 841 (**A**), UBC 811 (**B**), UBC 810 (**C**) and UBC 816 (**D**). The size of the amplified bands was calculated using 1kb DNA ladder (100–3000 bp) and the image has been converted to black and white and to negative.

The 10 ISSR primers used in the PCR screening (Table 5) produced for each gDNA sample 52 clear and reproducible bands and a total of 468 bands (number of bands X number of gDNA samples). The number of scorable bands for each primer varied from three (UBC 811) to seven (UBC 841) (Figure 5), with an average of 5.2 bands per primer. The ISSR-based bands were monomorphic and no genetic variation was detected between the eight randomly selected plantlets, which were produced from the artificial seeds after 12 weeks of cold storage, and the gardenia mother plant. These results corroborate previous reports on genetic stability of plantlets raised from cold-stored artificial seeds of the woody species *Rauvolfia tetraphylla* [23] and *Viburnum dentatum* [29]. ISSR markers, due to their simplicity and cost-effectiveness [43], have been successfully employed to assess the genetic stability of the regenerants from artificial seeds in various plant species such as *Sphagneticola calendulacea* [24], *Viburnum dentatum* [29], *Rauvolfia serpentina* [43], *Ocimum kilimandschricum* [78], and also in many other micropropagated plants [79,80,81,82,83,84].

## 4. Conclusions

The findings of the present study proved the feasibility of encapsulating gardenia shoot tips and nodal segments in alginate matrix, successful bead formation and storage at 4 °C for up to 12 weeks and subsequent retrieval at high rates of plantlets of genetic uniformity. The established artificial seed production protocol could be used for the short-term conservation of gardenia propagules, avoiding costly subcultures, for germplasm exchange and distribution, as well as for the large-scale production of genetically uniform planting material as an alternative method to standard micropropagation technique. This is the first report of the application of artificial seed technology in *Gardenia jasminoides* Ellis for the short-term cold conservation and subsequent regeneration of plantlets whose genetic stability was assessed using ISSR analysis.

## Figures and Tables

**Table 1:** Effect of MS nutrient medium addition in the encapsulation matrix (with 2.5% sodium alginate and 50 mM calcium chloride) on artificial seed germination of *G. jasminoides*, under light or in darkness, after 4 weeks of culture.

Treatments	Shoot Regeneration Response (%)	Number of Shoots/Bead	Length of Shoots (cm)
Light			
Alginate	46.7 ± 6.7b ^1,2^	2.1 ± 0.2b ^1,2^	0.4 ± 0.2ab ^1,2^
Alginate + MS	88.9 ± 3.7a	2.7 ± 0.2a	0.7 ± 0.2a
Darkness			
Alginate	13.3 ± 9.3d	1.4 ± 0.3c	0.2 ± 0.1b
Alginate + MS	28.9 ± 2.4c	2.0 ± 0.2b	0.3 ± 0.2ab
ANOVA (F)	36.89	15.00	2.67
Significance of F	*** ^3^	** ^3^	* ^3^

^1^ Mean (± SD) of 45 artificial seeds per treatment. ^2,3^ Different letters within columns indicate statistically significant differences according to Duncan’s multiple range test at *p* ≤ 0.05 (*). **, *** Significant at the 0.01 and 0.001 level, respectively.

**Table 2:** Effect of different concentrations of calcium chloride (CaCl_2_^.^2H_2_O), used with 2.5% sodium alginate, on bead morphology and hardness of *G. jasminoides*.

Concentration of Calcium Chloride (mM)	Grading of Bead Formation	Texture/Shape of Beads	Hardness οf Beads (N, Newton)
25	+ ^1^	Very delicate, fragile, indefinite shape	0.14 ± 0.01 ^2^
50	++	Solid, soft, translucent, uniform shape	0.20 ± 0.02
100	+++	Uniform, firm, translucent, globular shape	0.27 ± 0.02
200	++	Uniform, very hard, mostly globular- and tail-shaped	0.37 ± 0.03

^1^ + Poor quality, ++ good quality, +++ best quality, ^2^ mean (±SD) of 10 beads per calcium chloride concentration.

**Table 3:** Effect of different concentrations of calcium chloride (CaCl_2_^.^2H_2_O), used with 2.5% sodium alginate, on subsequent artificial seed germination of *G. jasminoides,* after 4 weeks of culture.

Concentration of Calcium Chloride (mM)	Shoot Regeneration Response (%)	Number of Shoots/Bead	Length of Shoots (cm)
25	20.0 ± 9.3c ^1,2^	1.8 ± 0.3b ^1,2^	0.6 ± 0.2b ^1,2^
50	40.0 ± 6.9b	2.3 ± 0.4ab	1.1 ± 0.2a
100	51.1 ± 2.2a	2.7 ± 0.3a	1.3 ± 0.3a
200	42.2 ± 4.5b	2.1 ± 0.2b	0.7 ± 0.2b
ANOVA (F)	5.40	3.61	8.17
Significance of F	* ^3^	* ^3^	** ^3^

^1^ Mean (±SD) of 45 artificial seeds per treatment. ^2,3^ Different letters within columns indicate statistically significant differences according to Duncan’s multiple range test at *p* ≤ 0.05 (*). ** Significant at the 0.01 level.

**Table 4:** Effect of storage at 4 °C for different time periods (weeks) on subsequent shoot formation of encapsulated and non-encapsulated shoot tip explants of *G. jasminoides*, on MS nutrient medium with 1 μM of IAA and 5 μM of BAP, after 4 weeks of culture.

Type of Explants	Storage Time (Weeks)	Number of Shoots	Length of Shoots (cm)
Non-encapsulated explants (naked)	0 (control)	2.1 ± 0.2a ^1,2^	0.6 ± 0.2a ^1,2^
	4	1.2 ± 0.2b	0.2 ± 0.1b
	8	1.2 ± 0.3b	0.2 ± 0.1b
	12	0.0 ± 0.0c	0.0 ± 0.0c
Encapsulated explants	0 (control)	2.5 ± 0.3a	0.6 ± 0.2a
	4	2.3 ± 0.3a	0.5 ± 0.2ab
	8	2.3 ± 0.3a	0.5 ± 0.2ab
	12	2.3 ± 0.2a	0.5 ± 0.1a
ANOVA (F)		49.47	4.30
Significance of F		*** ^3^	** ^3^

^1^ Mean (±SD) of 45 replicates (explants) per treatment, after 4 weeks of culture. ^2,3^ Different letters within columns indicate statistically significant differences according to Duncan’s multiple range test at *p* ≤ 0.05 (*). **, *** Significant at the 0.01 and 0.001 level, respectively.

**Table 5:** Code and sequence of 10 ISSR primers used to determine the genetic stability of *G. jasminoides* plantlets produced from encapsulated and cold-stored shoot tip explants.

Primer Code	Primer Sequence (5′-3′)	Annealing Temperature	Number of Bands
UBC 808	AGA GAG AGA GAG AGA GC	58 °C	6
UBC 809	AGA GAG AGA GAG AGA GG	58 °C	5
UBC 810	GAG AGA GAG AGA GAG AT	52 °C	5
UBC 811	GAG AGA GAG AGA GAG AC	54 °C	3
UBC 815	CTC TCT CTC TCT CTC TG	50 °C	6
UBC 816	CAC ACA CAC ACA CAC AT	54 °C	4
UBC 818	CAC ACA CAC ACA CAC AG	56 °C	6
UBC 821	GTG TGT GTG TGT GTG TT	56 °C	4
UBC 834	AGA GAG AGA GAG AGA GYT	56 °C	6
UBC 841	GAG AGA GAG AGA GAG AYC	50 °C	7

## Data Availability

Not applicable.
